# The Role of 20-HETE, COX, Thromboxane Receptors, and Blood Plasma Antioxidant Status in Vascular Relaxation of Copper-Nanoparticle-Fed WKY Rats

**DOI:** 10.3390/nu13113793

**Published:** 2021-10-26

**Authors:** Michał Majewski, Jerzy Juśkiewicz, Magdalena Krajewska-Włodarczyk, Leszek Gromadziński, Katarzyna Socha, Ewelina Cholewińska, Katarzyna Ognik

**Affiliations:** 1 Department of Pharmacology and Toxicology, UWM, 10-082 Olsztyn, Poland; 2 Division of Food Science, Institute of Animal Reproduction and Food Research, Polish Academy of Sciences, 10-748 Olsztyn, Poland; j.juskiewicz@pan.olsztyn.pl; 3 Department of Mental and Psychosomatic Diseases, Faculty of Medicine, UWM, 10-228 Olsztyn, Poland; magdalenakw@op.pl; 4 Department of Cardiology and Internal Medicine, Faculty of Medicine, UWM, 10-082 Olsztyn, Poland; leszek.gromadzinski@uwm.edu.pl; 5 Department of Bromatology, Medical University of Białystok, 15-222 Białystok, Poland; katarzyna.socha@umb.edu.pl; 6 Department of Biochemistry and Toxicology, Faculty of Biology, Animal Sciences and Bioeconomy, University of Life Sciences, 20-950 Lublin, Poland; ewelina.cholewinska@up.lublin.pl (E.C.); kasiaognik@poczta.fm (K.O.)

**Keywords:** aging, 20-HETE, furegrelate, HET0016, indomethacin, NS-398, SQ-29,548, thromboxane-A_2_

## Abstract

Recently, the addition of copper nanoparticles (NPs) in a daily diet (6.5 mg/kg) was studied in different animal models as a possible alternative to ionic forms. Male Wistar–Kyoto rats (24-week-old, *n* = 11) were fed with copper, either in the form of carbonate salt (Cu_6.5_) or metal-based copper NPs (NP_6.5_), for 8 weeks. The third group was fed with a half dose of each (NP_3.25_ + Cu_3.25_). The thoracic aorta and blood plasma was studied. Supplementation with NP_6.5_ decreased the Cu (×0.7), Cu/Zn-ratio (×0.6) and catalase (CAT, ×0.7), and increased Zn (×1.2) and superoxide dismutase (SOD, ×1.4). Meanwhile, NP_3.25_ + Cu_3.25_ decreased the Cu/Zn-ratio (×0.7), and CAT (×0.7), and increased the daily feed intake (×1.06). Preincubation with either the selective cyclooxygenase (COX)-2 inhibitor, or the non-selective COX-1/2 inhibitor attenuated vasodilation of rat thoracic aorta in the NP_6.5_ group exclusively. However, an increased vasodilator response was observed in the NP_6.5_ and NP_3.25_ + Cu_3.25_ group of rats after preincubation with an inhibitor of 20-hydroxyeicosatetraenoic acid (20-HETE) formation, and the thromboxane receptor (TP) antagonist. Significant differences were observed between the NP_6.5_ and NP_3.25_ + Cu_3.25_ groups of rats in: dietary intake, acetylcholine-induced vasodilation, and response to COX-inhibitors. Copper NPs in a standard daily dose had more significant effects on the mechanism(s) responsible for the utilization of reactive oxygen species in the blood plasma with the participation of prostanoids derived from COX-2 in the vascular relaxation. Dietary copper NPs in both doses modified vasodilation through the vasoconstrictor 20-HETE and the TP receptors.

## 1. Introduction

Copper fluctuations in a diet may have either pro- or antioxidant effects on animal or human health, dependent on the daily dose [1,2,3,4]. A high intake of copper (including the recommended daily dose) may induce oxidation of lipids and proteins in cells that are potentiated in situations of a high-risk susceptibility to toxic compounds, such as in diabetes mellitus or hypertension [5,6]. Many of the major enzymes of biological processes are influenced by copper intake, including Cu–Zn superoxide dismutase (SOD), cytochrome c oxidase, lysyl oxidase, L-ascorbate oxidase, monoamine oxidase, tyrosinase, and the enzymes of tryptophan degradation [7]. This may bring oxidative damage to lipids, proteins, and DNA, and result in neurodegenerative changes when dysregulated by either copper deficiency or its surplus [8,9].

Moreover, administration of copper nanoparticles (NPs) to animal feed, in a standard daily dose, may be of toxicological relevance due to its negative impact on animal health and the excretion of a large amount of this element into the environment and thus contamination [10]. Copper NPs induce a toxic effect by the increased production of free radicals, including hydroxyl radicals, hydrogen peroxides, and superoxide anions. The properties of metal NPs, including small size and high reactivity, increase their biological action, which may interfere with the physiological processes and the bioavailability of other macro- and microelements; therefore, the standard daily dose (6.5 mg/kg of diet) should probably be reduced to prevent increased toxicity [11].

As there are just a few studies regarding the safety of copper NPs in rats, and some of these results are controversial, we aimed to examine the influence of a standard 6.5 mg/kg dose of copper as NPs. In another group, the daily dose of NPs was reduced by half, to 3.25 mg/kg, and 3.25 mg/kg of copper carbonate was added instead. The third group was fed with 6.5 mg/kg of copper carbonate. The blood plasma antioxidant status was studied together with the participation of arachidonic acid metabolites in the vasodilator response of rat thoracic aorta to acetylcholine.

## 2. Materials and Methods

### 2.1. Drugs and Chemicals

Acetylcholine (chloride), indomethacin, noradrenaline (hydrochloride), and NS-398 were obtained from Sigma-Aldrich (St. Louise, MO, USA); copper as carbonate (purity ≥ 99%) from Poch (Gliwice, Poland); SQ-29,548, furegrelate, and HET0016 from Cayman Chemical (Ann Arbor, MI, USA). Stock solutions (10 mM) of these drugs were prepared in distilled water, except for noradrenaline, which was dissolved in NaCl (0.9%) + ascorbic acid (0.01% *w*/*v*) solution; HET0016, SQ-29,548, and indomethacin were dissolved in ethanol; 1400 W in methanol; and NS-398 in DMSO. The solvent concentration was less than 0.01% (*v*/*v*).

These solutions were stored at −20 °C, and appropriate dilutions were made in Krebs–Henseleit solution (KH in mM: NaCl 115; CaCl_2_ 2.5; KCl 4.6; KH_2_PO_4_ 1.2; MgSO_4_ 1.2; NaHCO_3_ 25; glucose 11.1) on the day of the experiment.

#### Metal-Based Copper Nanoparticles

Copper NPs (99.9% purity powder, 40–60 nm size, 12 m^2^/g SSA, spherical morphology, 0.19 g/cm^3^ bulk density, 8.9 g/cm^3^ true density) were purchased from Sky Spring Nanomaterials (Inc., Houston, TX, USA). Stock solution (5 g/L) was prepared in a rapeseed oil, and about 9% of NPs dissolved as Cu (II) ions; thus, the final suspension contained both NPs and released copper species. The zeta potential of the copper NP suspension was determined to be −30.3 mV (in PBS) and −38.3 mV (pH 5), and the size was 104 nm (in rapeseed oil) determined by dynamic light scattering with a Zetasizer Nano ZS (Malvern Instruments, Malvern, UK) [12].

### 2.2. Experimental Protocol

24-week-old normotensive Wistar–Kyoto (WKYs/NCrl) rats from Charles River (Sulzfeld, Germany) were allocated randomly to 3 groups (*n* = 11) and were fed individually for 8 weeks with experimental diets under standard laboratory conditions [1]. Exclusively male rats were studied, to enable comparison with the previous experimentations. The rats were fed with copper in a standard daily dose of 6.5 mg/kg, either as carbonate salt (Cu_6.5_) or metal NPs (NP_6.5_). Moreover, the third group (NP_3.25_ + Cu_3.25_) was fed with 3.25 mg of copper NPs plus 3.25 mg of copper carbonate. Animal pellets were prepared according to the American Institute of Nutrition, and the copper NPs were dissolved in pure rapeseed oil (5 g/L) and mixed with the diet weekly [1].

### 2.3. Experimental Procedures

Intraperitoneal injections of ketamine (100 mg/kg BW) and xylazine (10 mg/kg BW) were used for anesthesia, followed by exsanguination [13]. Blood was centrifuged at 3000× *g* for 10 min to separate the plasma, which was further stored at −80 °C until analysis. The thoracic aorta was dissected and kept in an ice-cold Krebs–Henseleit buffer.

### 2.4. Blood Analysis

Copper and zinc were measured by the ICP–OES method. Bovine liver was used as a certified reference material (NIST1577C) for quality control. The units are expressed as µM. Superoxide dismutase activity was measured with Ransod and Ransel diagnostic kits (Randox); meanwhile, catalase (CAT) was determined by the enzymatic decomposition of hydrogen peroxide into water and oxygen. Data are expressed in U/mL. The sums of reduced glutathione (GSH) and oxidized glutathione (GSSG) were determined using an enzymatic method (Cell Biolabs) [14]. The units are expressed as µM. The total antioxidant potency FRAP (Ferric Reducing Antioxidant Power) was measured colorimetrically at 594 nm through the reduction of Fe(III) to Fe(II) by antioxidants present in the sample. Data are expressed as µM. The malondialdehyde (MDA) generates the MDA–TBA adduct, which was quantified with a fluorometric assay kit (ab118970) at Ex/Em = 532/553 nm. Data are expressed as mM.

### 2.5. Vascular Reactivity Studies

Briefly, aortic rings of 4 mm length were mounted in a stagnant 5 mL Graz Tissue Bath System (Barcelona, Spain) under the pre-load tension of 1 cN and aerated with carbogen gas for 60 min (TAM-A Hugo Sachs Elektronik, March, Germany) [15,16]. The functional integrity of aortic rings was checked with high K^+^ (75 mM KCl) and ACh (10 μM). Next, aortic rings were incubated for 30 min with either the inducible nitric oxide synthase (iNOS) inhibitor (1 µM, 1400 W), the selective cyclooxygenase-2 (COX-2) inhibitor (10 μM, NS-398), the non-selective COX-1/2 inhibitor (10 μM, indomethacin), the inhibitor of 20-hydroxyeicosatetraenoic acid (20-HETE) formation (0.1 μM, HET0016), the thromboxane-A_2_ synthase inhibitor (1 μM, furegrelate), or the thromboxane-A_2_ receptor (TP) antagonist (1 μM, SQ-29,548), and contracted with noradrenaline (0.1 μM). Then, the cumulative doses of ACh (0.1 nM–10 μM) were added into the incubation chambers. Only one cumulative concentration–response curve (CCRC) was performed on each aortic ring.

### 2.6. Data Analysis and Statistics

Vascular relaxation was expressed as a percentage of the contractile response to noradrenaline NA (0.1 μM). The CCRCs were analyzed by a nonlinear regression model (log agonist vs. response), which determined the area under the curve (dAUC), the maximal response (E_max_, %), and the potency (pEC_50_ = −logEC_50_). The Gaussian distribution of residuals and homoscedasticity of variance were tested for all data with *n* = 11; “*n*” refers to independent values, not replicates. The group comparison was performed by either a parametric (*t*-test or ANOVA) or non-parametric test (Mann–Whitney U-test or Kruskal–Wallis test). Results are expressed as the means ± SEM (for CCRCs) and means ± SD. Due to the small group sizes (*n* < 12), outliers detected by Grubbs’ test were included in a data set [13]. The level of significance was when * *p* < 0.05.

## 3. Results

### 3.1. The General Characterization of WKY Rats

**Figure 1:**
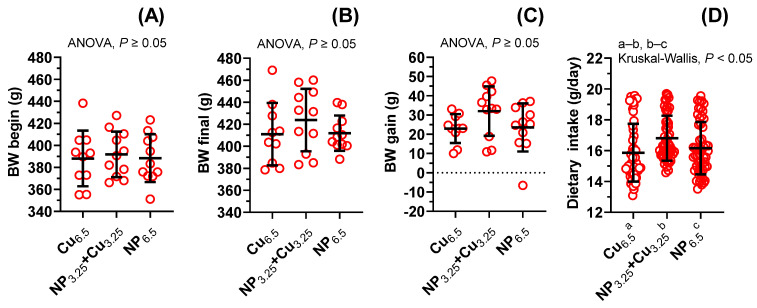
The influence of experimental diets on rat body weight (**A**–**C**), and daily feed intake (**D**). Values are expressed as means ± SD of *n* = 11 rats (**A**–**C**), and of *m* = 56 days of supplementation (**D**). NP_3.25_ + Cu_3.25_ increased by 1.06-fold the daily feed intake.

These results are presented in Figure 1A–D. Experimental supplementation with NP_6.5_ neither changed the body weight (BW) gain (×1.0, Figure 1C), nor the dietary intake (×1.02, Figure 1D). In the NP_3.25_ + Cu_3.25_ group of rats, BW gain was not changed in a significant way (×1.4, Figure 1C), contrary to the significant increase in daily feed intake (×1.06, *p* = 0.0008, Figure 1D). No significant difference was observed in the BW gain between NP_3.25_ + Cu_3.25_ and NP_6.5_ supplemented rats (×1.4, Figure 1C), as opposed to the increased daily feed intake (×1.04, *p* = 0.0462, Figure 1D).

### 3.2. Biomarkers of Oxidative Stress in the Blood Plasma

**Figure 2:**
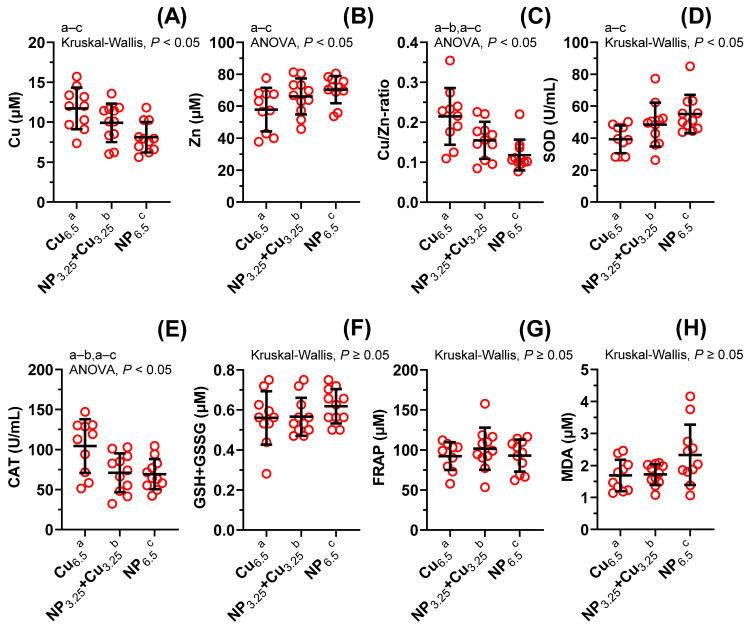
The influence of experimental diets on Cu, Zn content (**A**–**C**), and antioxidant mechanism (**D**–**H**) in blood plasma. Values are expressed as means ± SD of *n* = 11 rats. Supplementation with NP_6.5_ decreased the Cu (×0.7), Cu/Zn-ratio (×0.6), catalase (CAT, ×0.7), and increased Zn (×1.2), and superoxide dismutase (SOD, ×1.4). NP_3.25_ + Cu_3.25_ decreased the Cu/Zn-ratio (×0.7), and CAT (×0.7).

Supplementation with NP_6.5_ modified the Cu/Zn-ratio (×0.6, *p* = 0.007), Cu (×0.7, *p* = 0.0083), CAT (×0.7, *p* = 0.0134), Zn (×1.2, *p* = 0.0429), and SOD (×1.4, *p* = 0.0137); meanwhile, NP_3.25_ + Cu_3.25_ decreased the Cu/Zn-ratio (×0.7, *p* = 0.0397), and CAT (×0.7, *p* = 0.0134). NP_6.5_ did not change FRAP (×1.0), GSH + GSSG (×1.1) and MDA (×1.4); and NP_3.25_ + Cu_3.25_ did not change Cu (×0.8), MDA (×1.0), GSH + GSSG (×1.0), Zn (×1.1), FRAP (×1.1), and SOD (×1.2). There was no significant difference between NP_3.25_ + Cu_3.25_ and NP_6.5_ in the level of MDA (×0.7), GSH + GSSG (×0.9), SOD (×0.9), Zn (×0.9), CAT (×1.0), FRAP (×1.1), Cu (×1.2), and Cu/Zn-ratio (×1.3). Data are presented in Figure 2A–H.

### 3.3. Vascular Reactivity Studies

**Figure 3:**
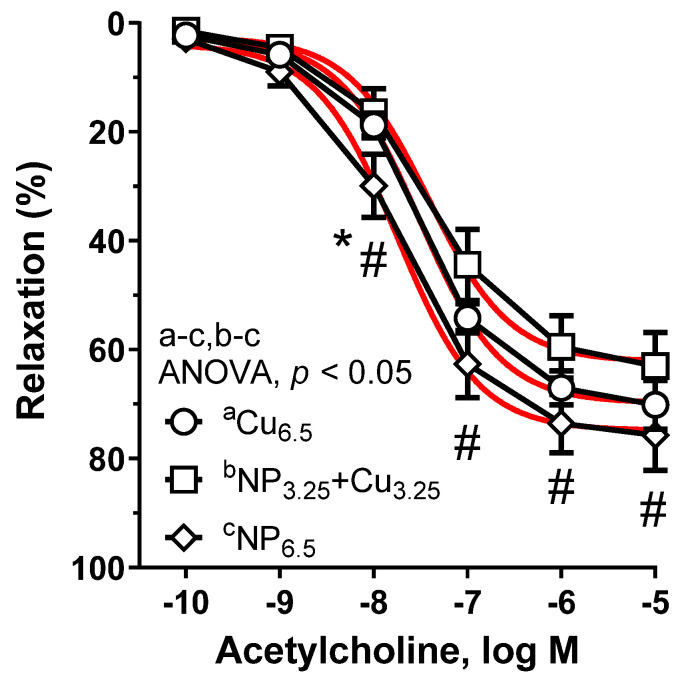
The relaxant response to acetylcholine in the isolated thoracic rings from rats supplemented with Cu_6.5_, NP_3.25_ + Cu_3.25_, and NP_6.5_. Results are means ± SEM, * compared to Cu_6.5_, ^#^ compared to NP_3.25_ + Cu_3.25_, *p* < 0.05 of *n* = 11 rats; ANOVA/Tukey’s. The red curve is the nonlinear regression model (log agonist vs. response). A significant change in the relaxant response was observed between the NP_3.25_ + Cu_3.25_ and NP_6.5_ groups of rats.

Neither NP_3.25_ + Cu_3.25_ (AUC: ×0.85) nor NP_6.5_ (AUC: ×1.18) changed the vasodilation compared to the control Cu_6.5_. However, there was a tendency to increased vasodilation at 10 nM of ACh in NP_6.5_ (see Figure 3). Moreover, significant change was observed in NP_3.25_ + Cu_3.25_ compared to NP_6.5_ (AUC: ×0.72). Preincubation with NS-398 diminished that response (between NP_3.25_ + Cu_3.25_ and NP_6.5_), which was completely abolished with indomethacin (see Table 1).

**Figure 4:**
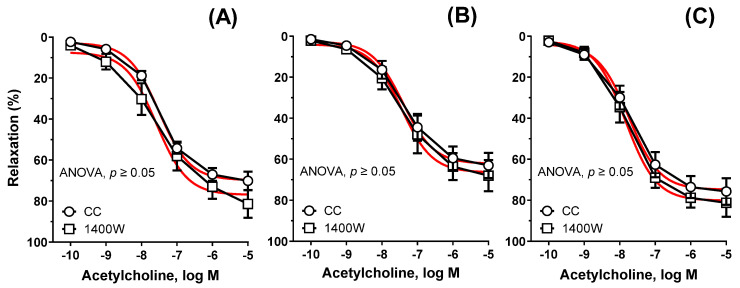
The influence of 1400 W on the relaxant response to acetylcholine. Aortic rings from rats supplemented with Cu_6.5_ (**A**), NP_3.25_ + Cu_3.25_ (**B**), and NP_6.5_ (**C**) were pre-incubated with the inducible nitric oxide synthase inhibitor (1400 W, 30 min, 1 μM). Results are means ± SEM, *p* > 0.05 of *n* = 5 rats; two-way ANOVA/Sidak’s. Preincubation with the selective iNOS inhibitor did not modify the vasodilation.

Preincubation with 1400 W (1 µM) did not modify the vasodilation in Cu_6.5_ (AUC: ×1.13), NP_3.25_ + Cu_3.25_ (AUC: ×1.04), and NP_6.5_ (AUC: ×0.92), see Figure 4A–C.

**Figure 5:**
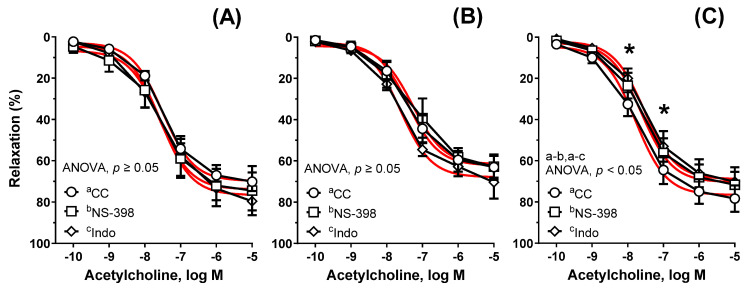
The influence of NS-398 and indomethacin (Indo) on the relaxant response to acetylcholine. Aortic rings from rats supplemented with Cu_6.5_ (**A**), NP_3.25_ + Cu_3.25_ (**B**), and NP_6.5_ (**C**) were pre-incubated with the selective cyclooxygenase-2 (COX-2) inhibitor (NS-398, 30 min, 10 μM) and the non-selective COX-1/2 inhibitor (Indo, 30 min, 10 μM). Results are means ± SEM, * *p* < 0.05 of *n* = 5 rats; ANOVA/Tukey’s. Preincubation with NS-398 and indomethacin attenuated vasodilation of rat thoracic aorta in the NP_6.5_ group exclusively.

Neither the selective COX-2 inhibitor (NS-398, 10 μM), nor the non-selective COX-1/2 inhibitor (indomethacin, 10 μM) changed the acetylcholine-induced response in the following groups of rats: Cu_6.5_ (AUC: ×1.09, and ×1.10, respectively, Figure 5A), and NP_3.25_ + Cu_3.25_ (AUC: ×0.90, and ×1.05, respectively, Figure 5B). However, a decreased response was observed in the NP_6.5_ group (×0.84, and ×0.81, respectively, Figure 5C). There was no significant difference between NS-398- and indomethacin-induced response in NP_6.5_ (AUC: ×1.04), opposite to some changes in NP_3.25_ + Cu_3.25_ (AUC: ×0.86).

**Figure 6:**
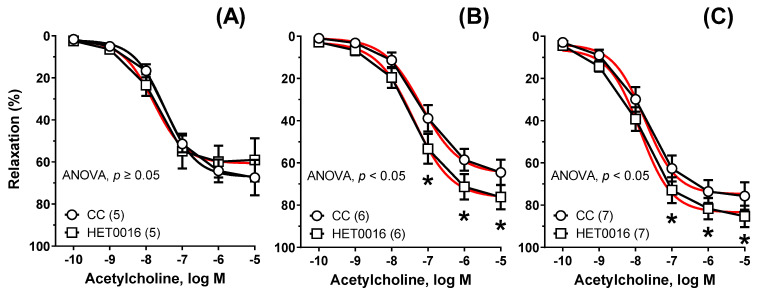
The influence of HET0016 on the relaxant response to acetylcholine. Aortic rings from rats supplemented with Cu_6.5_ (**A**), NP_3.25_ + Cu_3.25_ (**B**), and NP_6.5_ (**C**) were pre-incubated with an inhibitor of 20-HETE formation (HET0016, 30 min, 0.1 μM). Results are the means ± SEM, * *p* < 0.05 two-way ANOVA/Sidak’s. Number of animals is indicated in parenthesis. Preincubation with HET0016 potentiated vasodilation in NP_3.25_ + Cu_3.25_, and NP_6.5_ fed rats. This was not observed in the control group (Cu_6.5_).

Preincubation with HET0016 (0.1 μM) potentiated vasodilation in NP_3.25_ + Cu_3.25_ (AUC: ×1.39, Figure 6B), and NP_6.5_ (AUC: ×1.52, Figure 6C) fed rats. This was not observed in the control group Cu_6.5_ (AUC: ×1.02, Figure 6A).

**Figure 7:**
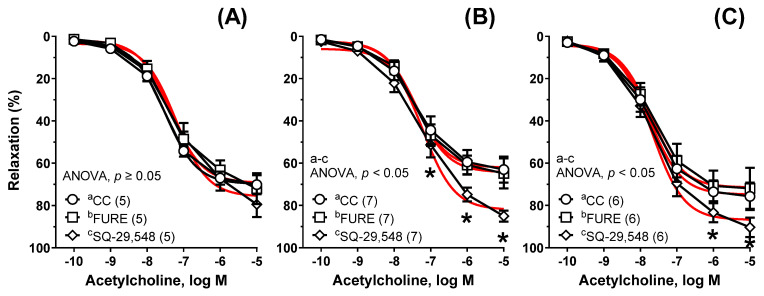
The influence of furegrelate and SQ-29,548 on the relaxant response to acetylcholine. Aortic rings from rats supplemented with Cu_6.5_ (**A**), NP_3.25_ + Cu_3.25_ (**B**), and NP_6.5_ (**C**) were pre-incubated with the thromboxane-A_2_ synthetase inhibitor (FURE, 30 min, 1 μM) and the thromboxane-A_2_ receptor antagonist (SQ-29,548, 30 min, 1 μM). Results are means ± SEM, * *p* < 0.05; ANOVA/Tukey’s. Number of animals is indicated in parenthesis. SQ-29,548 potentiated vasodilation in the NP_3.25_ + Cu_3.25_ and in the NP_6.5_ group of rats.

Neither the thromboxane-A_2_ synthase inhibitor (furegrelate, 1 μM) nor the TP antagonist (SQ-29,548, 1 μM) changed the acetylcholine-induced response in Cu_6.5_ (Figure 7A). However, in the NP_3.25_ + Cu_3.25_ (AUC: ×1.38, Figure 7B) and the NP_6.5_ (AUC: ×1.57, Figure 7C) groups of rats, SQ-29,548 potentiated vasodilation. Furegrelate did not modify that response (AUC: ×1.05, and ×1.03, respectively). Moreover, a significant increase was also observed between SQ-29,548 and furegrelate (AUC: ×1.31, and ×1.53, respectively) (Figure 7B,C).

The vasodilator response to acetylcholine is presented in Figure 3, Figure 4, Figure 5, Figure 6 and Figure 7. Results are expressed as E_max_ (%), pEC_50_ and AUC, see Table 1.

## 4. Discussion

Our previous studies revealed differences in the vascular tone regulation and the antioxidant status of rats supplemented with copper NPs (of 40–60 nm size) [1,2,12,17,18]. Of great importance is that in the previous experiments, both the age (either 4, 5, or 6 weeks) of Wistar Han IGS rats and the duration of feed intake (either 4 or 8 weeks) were what differentiated these studies from the one presented now, which can be described as 24 + 8 (24 weeks of age + 8 weeks of experimental feeding); and this was carried out on Wistar–Kyoto (WKY) rats, as a control for the spontaneously hypertensive rat (SHR) model which was also analyzed, but will be described elsewhere. Based on the previous results, antioxidant status and the participation of arachidonic acid metabolites were further investigated in the regulation of the vasodilator response induced by acetylcholine. Three different diets (i) standard with copper carbonate (Cu_6.5_), (ii) with metal copper NPs (NP_6.5_), and (iii) half dose of each (NP_3.25_ + Cu_3.25_) were prepared in the form of pellets, and given daily to rats in order to study the physiological properties of dietary copper NPs.

Experimental supplementation with NP_6.5_ neither modified the body weight nor the feed intake, which is in agreement with our previous results 4 + 8 (4 weeks of age + 8 weeks of experimental feeding), and 5 + 4 (5 weeks of age + 4 weeks of experimental feeding) [2,3,12,17]. However, in the NP_3.25_ + Cu_3.25_ group of rats, we observed a significant increase in the daily feed intake compared to Cu_6.5_ and NP_6.5_, and an increase of body weight gain (not significant), which might be explained by the higher feed intake, and may become significant when more rats per group are studied. The observed increase in feed intake is difficult to explain and merits further investigation. Experimental treatment with NP_6.5_ markedly reduced blood plasma Cu and increased Zn, which resulted in a decreased Cu/Zn-ratio. This is opposite to the 4 + 8 study, when Zn remained unchanged. However, in the same study, Cu and the Cu/Zn-ratio also decreased [18]. In the NP_3.25_ + Cu_3.25_ group of rats, the Cu/Zn-ratio decreased significantly, which was due to a decrease in Cu and an increase in Zn (both results were not significant). There was no statistically significant difference between NP_3.25_ + Cu_3.25_ and NP_6.5_ in the Cu, Zn, and Cu/Zn-ratio. We have now observed, for the first time, increased activity of SOD and decreased CAT in NP_6.5_ supplemented rats. This is contrary to our previous studies (4 + 8, and 6 + 8), when the activity of SOD was not modified and CAT increased [2,19]. However, another experiment from our research group (5 + 4) pointed to a decrease in CAT [12]. Increased SOD (result not significant) and decreased CAT were also observed for the NP_3.25_ + Cu_3.25_ group. Increased SOD indicates an effective means of scavenging superoxide anion, whereas decreased CAT points to possible enzyme depletion in response to the increased oxidative stress and intensified scavenging of hydrogen peroxide. Another enzyme of hydrogen peroxide degradation, glutathione peroxidase, was not modified in this study, and this is opposite to a significant decrease in the 5 + 4 study [12]. Copper in the form of NPs neither modified FRAP nor MDA. These findings are not entirely in agreement with our previous results (4 + 8 and 6 + 8), when FRAP increased [2,19], and MDA either increased (5 + 4 and 7 + 8) [1,12] or was not modified (4 + 8, and 6 + 8) [2,3,19]. We previously reported that replacing Cu_6.5_ with NP_6.5_ and reducing the dose (from a standard 6.5 mg/kg to a 3.25 mg/kg in the diet of rats 5 + 4) had particularly unfavorable effects on the respiratory system, causing adverse changes to the lungs. Surprisingly, these treatments also had a positive effect on the redox status of the liver and brain [20]. Moreover, the addition of copper NPs into the rat diet (5 + 4 and 7 + 8) reduced protein oxidation and nitration [1,12], as well as DNA oxidation and methylation. Meanwhile, lowering the daily dose increased the oxidation of proteins and DNA methylation [12]. In our study, neither NP_6.5_ nor NP_3.25_ + Cu_3.25_ modified the acetylcholine-induced vasodilation compared to Cu_6.5_ control. However there was a tendency to increased vasodilation in NP_6.5_ supplemented rats. This stays in agreement with the 4 + 8 study [2], and is in opposition to the 7 + 8 study, when NP_6.5_ potentiated the vasodilator response induced by acetylcholine in a significant way [1]. Surprisingly, in the present study, NP_6.5_ potentiated that response compared to NP_3.25_ + Cu_3.25_. Preincubation with NS-398 (COX-2 inhibitor) diminished that response, which was completely abolished with indomethacin (COX-1/2 inhibitor). These results suggest participation of COX-2 in NP-induced response, which is a dose dependent mechanism. In another experiment, conducted by Cendrowska-Pinkosz et al. [21], NP_6.5_ in the diet of rats did not change the acetylcholinesterase level (an enzyme that catalyzes the breakdown of acetylcholine) in the blood compared to Cu_6.5_ (7 + 8), so the observed changes might not be due to enzyme depletion nor surplus. Even though we did not currently report any changes in acetylcholine-induced vasodilation followed by NP_6.5_ and NP_3.25_ + Cu_3.25_ intake (compared to the control Cu_6.5_), the contribution of nitric oxide and arachidonic acid metabolites cannot be ruled out. We observed that iNOS inhibition with 1400 W did not modify that response in aortas from all three studied groups, which is contrary to the previous study (7 + 8), when the overproduction of NO from iNOS was engaged in vascular relaxation in the NP_6.5_ group of rats [1]. Considering that the sensitivity of the arteries to nitric oxide was not altered (study with an exogenous NO donor sodium nitroprusside) [2], arachidonic acid derivatives may also be responsible for the vascular tone regulation of copper NPs, as we suggested previously [18]. Preincubation with either COX-2 or COX-1/2 inhibitors attenuated the vasodilator response in NP_6.5_, indicating the involvement of a vasodilatory net effect of prostanoids origin from COX-2. This was neither observed for the control (Cu_6.5_) nor when the dose was reduced by half (in the NP_3.25_ + Cu_3.25_ group). The results with NP_6.5_ are contrary to our previous report (4 + 8), which had revealed a decreased sensitivity of the smooth muscles to prostanoids (no significant change in acetylcholine-induced response after COX-1/2 inhibition) [2]. Given these results, the participation of another vasoconstrictor agent in vascular relaxation was studied. 20-Hydroxyeicosatetraenoic acid (20-HETE) is a potent metabolite of arachidonic acid. This reaction is mediated by cytochrome P450 and can be further metabolized by COX to 20-hydroxy compounds. Preincubation with a 20-HETE inhibitor potentiated the vascular relaxation in both NP_6.5_ and NP_3.25_ + Cu_3.25_ groups, indicating that 20-HETE is involved in acetylcholine-induced relaxation. However, this was not observed in young rats (7 + 8) [1]. Finally, we analyzed the contribution of the potent vasoconstrictor thromboxane-A_2_ in the acetylcholine-induced response. However, the present results showed that thromboxane-A_2_ is not an important vasoconstrictor candidate engaged in the vascular tone regulation of supplemented rats (studies with the thromboxane-A_2_ synthesis inhibitor, furegrelate). Through the activation of thromboxane-A_2_ receptors (TP), prostacyclin, prostaglandins, isoprostanes, and 20-HETE participate in the endothelial dysfunction associated with cardiovascular risk factors [15]. In this study, when the TP were blocked with SQ-29,548, we observed increased vasodilation in both groups of copper NPs supplemented rats (NP_6.5_ and NP_3.25_ + Cu_3.25_). These data point towards a potent vasoconstrictor that acts on the TP, suggesting 20-HETE as a possible candidate. However, other vasoconstrictors should also be taken under consideration in further studies [16]. As there is an interplay between vasoconstrictor and vasodilator factors, vasodilators should also be analyzed; this will be done in another study.

These data point to some significant changes induced by metal copper NPs, which are age-dependent and are observed between younger (12-week-old) and older (32-week-old) male WKY rats. Indeed, age-related changes in blood vessels elasticity/stiffness might be exacerbated by daily diet [22]. Many recent studies have focused on dietary intervention to improve vascular health and delay the onset of vascular aging [23]. Dietary intervention may improve not only the vascular/cardiovascular impairment, but also vascular cognitive impairment and dementia. However, so far, only certain vitamins (vitamin E, folate), multi-nutrient formulations, and unsaturated fatty acids have shown some initial promise [24].

## 5. Conclusions

Our findings have shown that increased oxidative stress accompanies copper NP intake, which further modulates vascular relaxation with the participation of 20-HETE, through the thromboxane-A_2_ receptors. When a daily dose of copper NPs was decreased by half, the interplay between COX metabolites was also modified; however, the Cu/Zn-ratio and CAT remained unchanged compared to the higher dose. Further studies should concentrate on animals with metabolic disorders.

## Figures and Tables

**Table 1:** The influence of iNOS inhibitor (1400 W, 1.0 µM), the selective COX-2 inhibitor (NS-398, 10 µM), the non-selective COX-1/2 inhibitor (indomethacin, 10 µM), the inhibitor of 20-HETE formation (HET0016, 0.1 µM), the thromboxane-A_2_ synthase inhibitor (furegrelate, 1.0 µM), and the thromboxane receptor antagonist (SQ-29,548, 1.0 µM) on the vasorelaxant effects to acetylcholine of thoracic arteries from Wistar–Kyoto rats supplemented with Cu_6.5_, NP_3.25_ + Cu_3.25_ and NP_6.5_.

Group	Cu_6.5_				NP_3.25_ + Cu_3.25_				NP_6.5_			
	n	Emax (%)	pEC_50_	AUC	n	Emax (%)	pEC_50_	AUC	n	Emax (%)	pEC_50_	AUC
Control conditions	11	69.76	7.509	182.0	11	62.19	7.413	157.0	11	76.80 ^$^	7.748	214.3 ^$^
±SEM		1.899	0.087	12.73		3.460	0.172	25.01		3.294	0.147	25.10
+1400 W	5	74.95	7.581	205.9	5	66.40	7.454	173.2	5	80.15 ^$^	7.839	232.1 ^$^
±SEM		4.092	0.183	21.09		4.326	0.209	25.66		3.212	0.131	18.19
+HET0016	5	60.64	7.795	175.1	6	76.50 *^#^	7.386	190.3 *	7	88.44 *^#$^	7.874 ^$^	252.9 *^#$^
±SEM		4.180	0.227	22.66		4.714	0.165	19.81		2.919	0.120	20.99
+SQ-29,548	5	75.77	7.210	176.2	7	82.08 *	7.222	199.0 *	6	86.82 *^#^	7.683 ^#$^	241.4 *^#^
±SEM		3.725	0.139	17.27		2.855	0.102	15.66		2.929	0.112	17.28
+FURE	5	69.25	7.360	167.1	7	64.34	7.387	159.7	6	71.47	7.711	202.2
±SEM		2.888	0.124	12.77		3.030	0.143	16.53		4.186	0.195	27.60
+NS-398	5	73.82	7.582	207.5	5	61.61 ^#^	7.277	152.7 ^#^	5	70.36	7.624 ^$^	190.9 *
±SEM		5.746	0.267	29.95		4.113	0.200	20.22		3.652	0.170	24.58
+INDO	5	76.61	7.564	206.6	5	67.88	7.618	182.8	5	68.88	7.540	179.8 *
±SEM		4.008	0.173	21.78		3.088	0.158	13.54		3.772	0.174	22.93

Values are based on the concentration–response curves shown in Figure 3, Figure 4, Figure 5, Figure 6 and Figure 7. Data are expressed as means ± SEM where n represents the number of animals. * *p* < 0.05 compared with the control conditions, ^#^ *p* < 0.05 compared with the Cu_6.5_ group, ^$^ *p* < 0.05 compared with the NP_3.25_ + Cu_3.25_ group as determined by one-way ANOVA followed by Tukey’s post hoc test.

## Data Availability

Data supporting reported results can be found in Supplementary Materials.

## Supplementary Materials

The following are available online at https://www.mdpi.com/article/10.3390/nu13113793/s1, Table S1: Experimental results (means with SD).

## References

[1] Majewski M., Lis B., Olas B., Ognik K., Juśkiewicz J. (2020). Dietary supplementation with copper nanoparticles influences the markers of oxidative stress and modulates vasodilation of thoracic arteries in young Wistar rats. PLoS ONE.

[2] Majewski M., Ognik K., Juśkiewicz J. (2019). Copper nanoparticles modify the blood plasma antioxidant status and modulate the vascular mechanisms with nitric oxide and prostanoids involved in Wistar rats. Pharmacol. Rep..

[3] Majewski M., Ognik K., Juśkiewicz J. (2019). The interaction between resveratrol and two forms of copper as carbonate and nanoparticles on antioxidant mechanisms and vascular function in Wistar rats. Pharmacol. Rep..

[4] Tang H., Xu M., Luo J., Zhao L., Ye G., Shi F., Lv C., Chen H., Wang Y., Li Y. (2019). Liver toxicity assessments in rats following sub-chronic oral exposure to copper nanoparticles. Environ. Sci. Eur..

[5] Galhardi C.M., Diniz Y.S., Faine L.A., Rodrigues H.G., Burneiko R.C.M., Ribas B.O., Novelli E.L. (2004). Toxicity of copper intake: Lipid profile, oxidative stress and susceptibility to renal dysfunction. Food Chem. Toxicol..

[6] Majewski M., Jurgoński A., Fotschki B., Juśkiewicz J. (2018). The toxic effects of monosodium glutamate (MSG)—The involvement of nitric oxide, prostanoids and potassium channels in the reactivity of thoracic arteries in MSG-obese rats. Toxicol. Appl. Pharmacol..

[7] Majewski M., Kozlowska A., Thoene M., Lepiarczyk E., Grzegorzewski W.J. (2016). Overview of the role of vitamins and minerals on the kynurenine pathway in health and disease. J. Physiol. Pharmacol..

[8] Majewski M., Ognik K., Thoene M., Rawicka A., Juśkiewicz J. (2020). Resveratrol modulates the blood plasma levels of Cu and Zn, the antioxidant status and the vascular response of thoracic arteries in copper deficient Wistar rats. Toxicol. Appl. Pharmacol..

[9] Majewski M., Kasica N., Jakimiuk A., Podlasz P. (2018). Toxicity and cardiac effects of acute exposure to tryptophan metabolites on the kynurenine pathway in early developing zebrafish (Danio rerio) embryos. Toxicol. Appl. Pharmacol..

[10] Luo J., Hao S., Zhao L., Shi F., Ye G., He C., Lin J., Zhang W., Liang H., Wang X. (2020). Oral exposure of pregnant rats to copper nanoparticles caused nutritional imbalance and liver dysfunction in fetus. Ecotoxicol. Environ. Saf..

[11] Jankowski J., Ognik K., Kozłowski K., Stępniowska A., Zduńczyk Z. (2019). Effect of different levels and sources of dietary copper, zinc and manganese on the performance and immune and redox status of Turkeys. Animals.

[12] Ognik K., Cholewińska E., Juśkiewicz J., Zduńczyk Z., Tutaj K., Szlązak R. (2019). The effect of copper nanoparticles and copper (II) salt on redox reactions and epigenetic changes in a rat model. J. Anim. Physiol. Anim. Nutr. Berl..

[13] Majewski M., Lis B., Juśkiewicz J., Ognik K., Jedrejek D., Stochmal A., Olas B. (2021). The composition and vascular/antioxidant properties of Taraxacum officinale flower water syrup in a normal-fat diet using an obese rat model. J. Ethnopharmacol..

[14] Żary-Sikorska E., Fotschki B., Jurgoński A., Kosmala M., Milala J., Kołodziejczyk K., Majewski M., Ognik K., Juśkiewicz J. (2020). Protective effects of a strawberry ellagitannin-rich extract against pro-oxidative and pro-inflammatory dysfunctions induced by a high-fat diet in a rat model. Molecules.

[15] Majewski M., Kucharczyk E., Kaliszan R., Markuszewski M., Fotschki B., Juskiewicz J., Borkowska-Sztachańska M., Ognik K. (2020). The characterization of ground raspberry seeds and the physiological response to supplementation in hypertensive and normotensive rats. Nutrients.

[16] Majewski M., Lepczyńska M., Dzika E., Grzegorzewski W., Markiewicz W., Mendel M., Chłopecka M. (2019). Evaluation of the time stability of aortic rings in young wistar rats during an eight-hour incubation period. J. Elementol..

[17] Majewski M., Ognik K., Zdunczyk P., Juskiewicz J. (2017). Effect of dietary copper nanoparticles versus one copper (II) salt: Analysis of vasoreactivity in a rat model. Pharmacol. Rep..

[18] Majewski M., Ognik K., Juśkiewicz J. (2019). Copper nanoparticles enhance vascular contraction induced by prostaglandin F2-alpha and decrease the blood plasma cu-zn ratio in wistar rats. J. Elementol..

[19] Majewski M., Ognik K., Juśkiewicz J. (2020). The antioxidant status, lipid profile, and modulation of vascular function by fish oil supplementation in nano-copper and copper carbonate fed Wistar rats. J. Funct. Foods.

[20] Ognik K., Cholewińska E., Tutaj K., Cendrowska-Pinkosz M., Dworzański W., Dworzańska A., Juśkiewicz J. (2020). The effect of the source and dosage of dietary Cu on redox status in rat tissues. J. Anim. Physiol. Anim. Nutr. Berl..

[21] Cendrowska-Pinkosz M., Krauze M., Juśkiewicz J., Ognik K. (2021). The effect of the use of copper carbonate and copper nanoparticles in the diet of rats on the level of β-amyloid and acetylcholinesterase in selected organs. J. Trace Elem. Med. Biol..

[22] Logvinov S.V., Naryzhnaya N.V., Kurbatov B.K., Gorbunov A.S., Birulina Y.G., Maslov L.L., Oeltgen P.R. (2021). High carbohydrate high fat diet causes arterial hypertension and histological changes in the aortic wall in aged rats: The involvement of connective tissue growth factors and fibronectin. Exp. Gerontol..

[23] Balasubramanian P., DelFavero J., Ungvari A., Papp M., Tarantini A., Price N., de Cabo R., Tarantini S. (2020). Time-restricted feeding (TRF) for prevention of age-related vascular cognitive impairment and dementia. Ageing Res. Rev..

[24] Vlachos G.S., Scarmeas N. (2019). Dietary interventions in mild cognitive impairment and dementia. Dialogues Clin. Neurosci..

