# Supplementary material for: The Role of 20-HETE, COX, Thromboxane Receptors, and Blood Plasma Antioxidant Status in Vascular Relaxation of Copper-Nanoparticle-Fed WKY Rats
Source: Nutrients. 2021 Oct 26;13(11):3793. doi: 10.3390/nu13113793 (PMC8623823; doi:10.3390/nu13113793)
Supplement: Supplementary file 1 [file nutrients-13-03793-s001.zip › nutrients-1415885-supplementary.pdf]

Table S1. Experimental results (means with SD).

Index	Mean ± SD		
	**$Cu_{6.5}$**	**$(NP+Cu)_{6.5}$**	**$NP_{6.5}$**
Initial body weight, g	388	392	388
±	25.3	20.7	21.8
Final body weight, g	411	424	412
±	28.4	28.4	16
Body weight gain, g	23	32	23.5
±	7.46	12.8	12.5
Dietary intake, g/day[1]	15.90	16.80	16.20
±	1.87	1.45	1.7
Cu, µM/L	11.70	9.920	8.120
±	2.600	2.410	1.920
Zn, µM/L	57.80	66.00	70.30
±	13.60	11.30	8.550
Cu/Zn ratio	0.214	0.155	0.118
±	0.071	0.047	0.038
SOD, U/mL	39.30	48.50	55.10
±	8.68	13.70	12.00
CAT, U/mL	104.0	71.00	69.40
±	33.60	24.30	19.00
GSH+GSSG, µmol/L	0.559	0.565	0.618
±	0.134	0.095	0.087
FRAP, µmol/L	92.20	102.00	92.80
±	17.20	26.30	19.90
MDA, µmol/L	1.690	1.720	2.330
±	0.492	0.327	0.942

[1] daily dietary intake during 8 weeks of supplementation, n = 56
